# Association of Perceived Stress with Atopic Dermatitis in Adults: A Population-Based Study in Korea

**DOI:** 10.3390/ijerph13080760

**Published:** 2016-07-27

**Authors:** Hyejin Park, Kisok Kim

**Affiliations:** 1 Department of International Medical Management, Catholic University of Daegu, Kyungbuk 38430, Korea; hjpark@cu.ac.kr; 2 College of Pharmacy, Keimyung University, Daegu 42601, Korea

**Keywords:** atopic dermatitis, perceived stress, adult population, KNHANES

## Abstract

Atopic dermatitis (AD) is a widely prevalent skin disease that affects both children and adults. The aim of the study was to assess the association of perceived stress (single-item, self-reported) with AD (self-reported) in a sample of Korean adults using a cross-sectional research design. A cross-sectional study was conducted using data from 33,018 adults aged 20 years and older collected in the 2007–2012 Korea National Health and Nutrition Examination Surveys (KNHANES). An increased level of self-reported stress was positively associated with an increased prevalence of AD in Korean adults (*p* for trend <0.001). After adjusting for covariates, the odds ratios (ORs) of AD among participants reporting high and very high levels of stress were 1.81 (95% confidence interval (CI): 1.22, 2.67) and 2.17 (95% CI: 1.38, 3.42), respectively, compared with those who reported low levels of stress. This study found a statistically significant association between perceived stress and AD among Korean adults.

## 1. Introduction

Atopic dermatitis (AD), a chronic inflammatory skin disease characterized by eczematous lesions and intense itching, is a global public health concern not only in children but also in adults [1,2,3]. AD is caused by a genetic predisposition and environmental factors, including null mutations in filaggrin (*FLG*), climate, urban living, and diet [4,5]. However, the major etiologic factor remains unknown. 

AD may be associated with psychological problems, such as anxiety, depression, sleep disorders, and personality disorders [6,7]. Of the many factors related to AD, psychological stress is thought to be particularly important [6,8]. Furthermore, numerous studies have demonstrated that psychological stress has a significant impact on skin inflammation [9].

Several studies have demonstrated a relationship between stress and AD [10,11]. Most have reported that psychological stress exacerbates AD, and patients with AD experience a worsening of the skin after exposure to stress [12,13,14]. Considering the mechanism, psychological stress produces various neuroendocrine mediators, including adrenocorticotropin, β-endorphin, catecholamines, and cortisol, which in turn activates local neurogenic inflammation and disrupts skin barrier function [15]. Although the association between AD and mental health comorbidities in children and adolescents has been well established [16], there are only a few recent population-based studies of AD for adults [17,18].

Although AD most frequently starts in early infancy and persists during adolescence, it is also highly prevalent in adults [19]. However, relatively little is known about the association between AD and psychological stress in adults, particularly in Koreans. The psychological stress response was conceptualized as perceived stress in this study. In the present study, the self-rated perceived stress level was used as a measure of participants’ perception regarding how much stress they are under in everyday life. Therefore, the goal of this study was to determine the association between perceived stress and AD in Korean adults by using data obtained from the 2007–2012 Korea National Health and Nutrition Examination Survey (KNHANES), a nationally representative survey conducted in the Republic of Korea.

## 2. Methods 

### 2.1. Study Population

This study was based on data from the fourth and fifth waves (2007–2012) of KNHANES, provided by the Korea Centers for Disease Control and Prevention. Samples from KNHANES were selected using a stratified, multi-stage, cluster-sampling design with proportional allocation based on the National Census Registry. Detailed information on the survey design and sampling procedures has been reported elsewhere [20]. Using weighted data from the 2007–2012 KNHANES databases, 33,018 adults aged 20 years and older who had no missing responses on the questionnaire were included in this study.

### 2.2. Data Collection

KNHANES included questions on demographic and socioeconomic characteristics, smoking and drinking habits, and psychological health. Information regarding AD and stress was obtained by a self-administered questionnaire. The criterion for AD was self-reported current AD. The level of perceived stress was measured using the following question: “How much stress do you usually feel?” Four response options were provided: “only a little”, “to some extent”, “to a great extent”, and “to a very great extent”, which were labeled as low, medium, high, and very high levels of perceived stress, respectively. The KNHANES program is approved by the KNHANES Institutional Review Board (IRB) (# KCDC-2007-02CON-04-P, KCDC-2008-04EXP-01-C, KCDC-2009-01CON-03-2C, KCDC-2010-02CON-21-C, KCDC-2011-02CON-06-C, KCDC-2012-01EXP-01-2C) and was conducted in accordance with the Ethical Principles for Medical Research Involving Human Subjects, as defined by the Helsinki Declaration. All study participants provided informed written consent.

### 2.3. Statistical Analyses

As appropriate, the frequency, mean, and 95% confidence intervals (CIs) were calculated to describe the demographic characteristics according to categories of AD or perceived stress. We evaluated differences in categorical variables using the Mantel-Haenszel chi-square test, and differences among groups in continuous variables were examined using the linear trend test. Logistic regression models were used to estimate the odds ratio (OR) and 95% CIs for AD among participants who reported medium to very high stress compared with the reference group (those who reported low stress). The presence of a linear trend was evaluated by defining a linear contrast in each of the linear and logistic regression models. All statistical analyses were conducted using SAS v9.3 (SAS Institute, Cary, NC, USA). Statistical analyses accounted for the survey design, and appropriate procedures in SAS, such as surveyfreq and surveylogistic, were used with weighted data. 

## 3. Results

This study included 33,018 adults aged 20 years and older; 607 (1.8%) reported having AD. The prevalence rates of AD did not differ significantly according to sex, BMI (body mass index), income, and alcohol consumption. However, age, educational level, and cigarette smoking were significantly associated with prevalence of AD (Table 1). The mean age of participants with and without AD was 41.5 year and 50.4 year, respectively.

The demographic characteristics of the study population are presented in Table 2 by severity of self-reported stress. The severity of self-reported stress was significantly correlated with sex, age, educational level, cigarette smoking, and alcohol consumption (*p* for trend <0.001).

Table 3 shows the population-weighted prevalence and ORs (odds ratio) of AD by self-reported stress. A significant positive trend was observed between the level of stress and AD prevalence (*p* for trend <0.001), with 1.26% of adults who felt low levels of stress reporting AD compared with 3.47% of those who felt very high levels of stress. Additionally, the crude and adjusted ORs for AD were positively correlated with increased self-reported stress (*p* for trend <0.001). In the full adjusted model (model 3), the ORs for AD were 1.22 (95% CI: 0.86, 1.73) among those who reported medium levels of stress, 1.81 (95% CI: 1.22, 2.67) among those who reported high levels of stress, and 2.17 (95% CI: 1.38, 3.42) among those who reported very high levels of stress compared with those who reported low levels of stress.

## 4. Discussion

This nationwide population-based study of Korean adults found that 1.8% of participants had AD, which is quite similar to the results of a recent study showing a 1.88% of AD prevalence in Korean adults [21]. This finding is consistent with previous reports that AD is one of the most common skin diseases, with a prevalence of 1% to 3% in adults [22,23]. In previous population-based studies, the prevalence of AD was 1.2% in Korean males aged around 20 years, whereas the prevalence of AD among adolescents aged 12–18 years was 22.4% [24,25]. In this study, we found that females were more stressed than males, which is consistent with a report that in Korea, women are more likely to experience stress and depression than men [26]. In addition, psychological stress was correlated with smoking cigarettes and drinking alcohol. Similar to our results, many experimental and epidemiological studies support the relationships between stress and such behaviors [27,28].

The results of this study show that psychological stress is related to AD in Korean adults. We found that participants with higher levels of stress had an increased prevalence of AD compared with those who had lower levels of perceived stress. Moreover, the ORs of AD were significantly increased with increasing psychological stress even after controlling for potential confounders. Overall, higher self-reported levels of psychological stress were associated with higher odds of AD and the association was robust after adjusting for multiple sociodemographic and lifestyle factors. Compared with those in children or adolescents, few epidemiological studies have been conducted on relationships between AD and psychological stress in adults [29]. However, several studies reported that adult patients with AD have significantly higher levels of psychological stress, including depression, than adults without AD [29,30]. Notably, a recent study reported a strong association between psychological stress and AD among young Korean males around 20 years old [24]. These findings are consistent with the results of the present study showing that psychological stress is strongly associated with AD in adults. 

The mechanisms underlying the association between psychological stress and AD are not fully understood. One possible mechanism underpinning these associations involves the role of cytokines as peripheral inflammatory mediators that modulate bidirectional communication between systemic inflammatory responses and brain functions, such as stress and depression [31]. Alternatively, psychological stress may lead to AD-relevant immunological changes via activation of neuroendocrine pathways including the hypothalamic-pituitary-adrenal (HPA) axis and sympatho-adrenomedullary system or via direct nervous inputs [32]. In addition, animal studies have demonstrated that neurotrophin- and neuropeptide-dependent neurogenic inflammation exerts stress-induced aggravation of allergic flares [33].

The present study has several limitations. As a result of the cross-sectional design, we were unable to establish a temporal relationship between AD and psychological stress; therefore, causality could not be determined. We relied on self-reports to assess stress levels, which may have led to misclassification and measurement errors. However, self-reporting of perceived stress provides an appropriate measure of the actual levels of stress experienced by individuals [34]. Additionally, information about AD was obtained via self-reports rather than direct diagnosis, which may have led to reporting bias. Despite these limitations, this is the first study assessing the association between psychological stress and AD in Korean adults using nationally representative data. Thus, given the advantages of systematic sampling, the results of this study can be generalized to all Korean adults. Future research, including cohort studies, will be important for assessing the causal relationship between psychological stress and the pathogenesis or severity of AD in adult populations.

## 5. Conclusions 

In this study, we found that perceived stress was strongly associated with AD in Korean adults. Given that stress interferes with several physiological and pathological processes, our results emphasize that stress may play an important role in the etiology and prognosis of AD. Because AD affects the physical, psychological, psychosocial, and occupational outlook of the patient, at great cost not only to the patient but also to society, broad social policies and interventions are required to mitigate psychological stress in adults. In addition, an assessment of psychological factors would be important to identify a high-risk subpopulation, which would allow earlier intervention and thereby prevent the onset and exacerbation of AD.

## Figures and Tables

**Table 1:** Distribution of demographic characteristics by prevalence of atopic dermatitis among Korean adults ≥20 years of age (*n* (%)).

Characteristics	No.	AD (% within Group)	No AD (% within Group)	*p*-Value *^a^*
Total	33,018	607 (100)	32,411 (100)	
Sex				0.685
Male	13,985	262 (43.2)	13,723 (42.3)	
Female	19,033	345 (56.8)	18,688 (57.7)	
Age (years)				<0.001
20–39	10,136	331 (54.5)	9805 (30.3)	
40–59	12,316	170 (28.0)	12,146 (37.5)	
≥60	10,566	106 (17.5)	10,460 (32.3)	
BMI				0.416
<18.5	1484	36 (5.9)	1448 (4.5)	
18.5–22.9	13,123	241 (39.7)	12,882 (39.8)	
23–27.4	14,457	255 (42.0)	14,202 (43.8)	
≥27.5	3954	75 (12.4)	3879 (12.0)	
Education				<0.001
<high school	12,408	142 (23.4)	12,266 (37.9)	
high school	9440	153 (25.2)	9287 (28.7)	
>high school	11,170	312 (51.4)	10,858 (33.5)	
Income				0.500
Q1 (lowest)	8111	154 (25.4)	7957 (24.6)	
Q2	8294	139 (22.9)	8155 (25.2)	
Q3	8327	147 (24.2)	8180 (25.2)	
Q4 (highest)	8286	167 (27.5)	8119 (25.1)	
Cigarette smoking				0.001
Yes	10,175	226 (37.2)	9949 (30.7)	
No	22,843	381 (62.8)	22,462 (69.3)	
Alcohol drinking				0.109
Yes	17,213	336 (55.4)	16,877 (52.1)	
No	15,805	271 (44.7)	15,534 (47.9)	

Abbreviations: AD, atopic dermatitis; BMI, body mass index. ***^a^*** *p*-Values were calculated using a Mantel-Haenzel chi-square test.

**Table 2:** Demographic characteristics by categories of self-reported stress.

Characteristics	Stress				*p*-Value *^a^*
	Low (*n* = 5527)	Medium (*n* = 18,472)	High (*n* = 7442)	Very High (*n* = 1577)	
AD, n (%)	61 (1.10)	312 (1.69)	188 (2.53)	46 (2.92)	<0.001
Gender, % female	53.4	56.6	62.2	63.3	<0.001
Mean age, year (95% CI)	59.5 (59.1, 59.9)	48.8 (48.6, 49.0)	47.2 (46.8, 47.6)	49.3 (48.5, 50.1)	<0.001
BMI, kg/m^2^ (95% CI)	23.9 (23.8, 24.0)	23.6 (23.6, 23.6)	23.7 (23.6, 23.7)	23.9 (23.7, 24.1)	0.753
Education > high school, %	21.6	35.7	38.7	31.1	<0.001
Income, US$/month (95% CI)	2502 (2302, 2702)	3247 (3132, 3362)	3104 (2918, 3291)	2805 (2519, 3091)	0.315
Cigarette smoking, %	30.3	29.8	32.8	35.0	<0.001
Alcohol drinking, %	43.8	53.8	54.5	50.5	<0.001

Abbreviations: AD, atopic dermatitis; CI, confidence interval; BMI, body mass index. ***^a^*** *p*-Values were calculated using a Mantel-Haenzel chi-square test for categorical variables or by a linear trend test for continuous variables.

**Table 3:** Weighted prevalence and odds ratios (95% CI) of atopic dermatitis by self-reported stress.

Stress	Low	Medium	High	Very High	*p* for Trend
Prevalence, %	1.26	1.91	3.02	3.47	<0.001
OR (95% CI)					
Model 1	1.00 (reference)	1.52 (1.09, 2.14)	2.43 (1.68, 3.53)	2.81 (1.80, 4.38)	<0.001
Model 2	1.00 (reference)	1.20 (0.85, 1.69)	1.80 (1.22, 2.65)	2.18 (1.39, 3.43)	<0.001
Model 3	1.00 (reference)	1.22 (0.86, 1.73)	1.81 (1.22, 2.67)	2.17 (1.38, 3.42)	<0.001

Abbreviations: OR, odds ratio; CI, confidence interval. Model 1: crude model. Model 2: adjusted for sex and age. Model 3: adjusted for sex, age, BMI, education, income, cigarette smoking, and alcohol consumption.
